# A monoclinic polymorph of 1,2-bis­[(1-methyl-1*H*-tetra­zol-5-yl)sulfan­yl]ethane (BMTTE)

**DOI:** 10.1107/S205698901701341X

**Published:** 2017-09-25

**Authors:** Saray Argibay-Otero, Olaya Gómez-Paz, Rosa Carballo

**Affiliations:** a Departamento de Química Inorgánica, Facultade de Química, Instituto de Investigación Sanitaria Galicia Sur – Universidade de Vigo, Campus Universitario, E-36310 Vigo, Galicia, Spain

**Keywords:** crystal structure, polymorph, tetra­zole-containing compounds, hydrogen bonding, π–π inter­actions

## Abstract

The synthesis and crystal structure of a monoclinic (*P*2_1_/*c*) polymorph of the title compound, C_6_H_10_S_2_N_8_, are reported. The mol­ecule has pseudo-twofold rotational symmetry, with the tetra­zole rings being inclined to one another by 5.50 (6)°. In the crystal, mol­ecules are linked by C—H⋯N hydrogen bonds, forming chains propagating along [101] and enclosing *R*_2_^2^(20) ring motifs. The chains are linked by offset π–π inter­actions involving the tetra­zole rings [inter­centroid distances vary from 3.3567 (7) to 3.4227 (7) Å], forming layers parallel to the *ac* plane. The crystal structure of the triclinic polymorph (*P* ) has been described previously [Li *et al.* (2011 ▸). *Acta Cryst.* E**67**, o1669].

## Abstract 2

The mol­ecular and crystal structures of a monoclinic polymorph of 1,2-bis­[(1-methyl-1*H*-tetra­zol-5-yl)sulfan­yl]ethane (BMTTE) are described.

## Chemical context   

Organic compounds such as the title compound (BMTTE) are frequently used as flexible ligands for the preparation of coordination polymers (Wang *et al.*, 2010 ▸). A triclinic polymorph of the title compound has been described previously by Li *et al.*, (2011 ▸). Here we describe the spectroscopic characterization and crystal structure of a new monoclinic polymorph of BMTTE, obtained by recrystallization and slow evaporation from a solution in CH_3_CN. Such compounds have been used in coordination chemistry (Zhao *et al.*, 2008 ▸) and in materials design (Wang *et al.*, 2009 ▸, 2010 ▸).
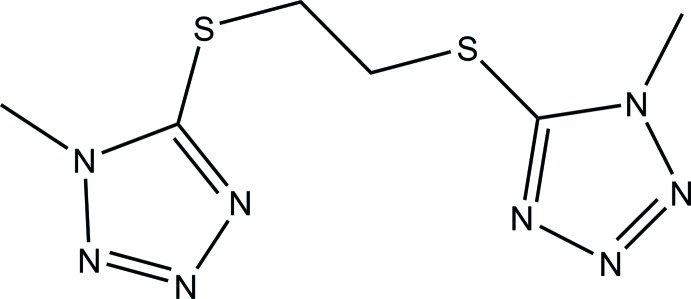


## Structural commentary   

**Figure 1:**
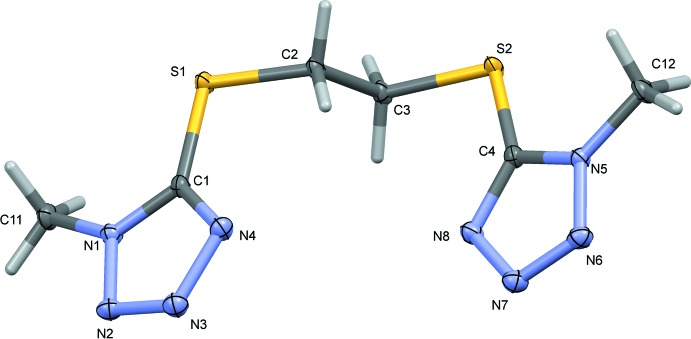
Mol­ecular structure of the title compound, the monoclinic polymorph of BMTTE, with atom labelling. Displacement ellipsoids are drawn at the 50% probability level.

**Figure 2:**
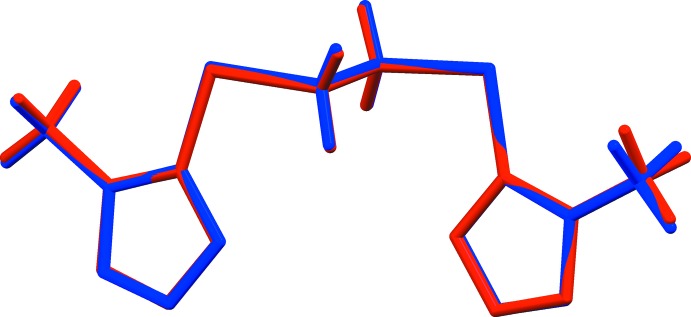
A mol­ecular structure overlap (*Mercury*; Macrae *et al.*, 2008 ▸) of the title monoclinic polymorph of BMTTE (blue) and the triclinic polymorph (red; Li *et al.*, 2011 ▸).

The mol­ecule structure of the title compound, Fig. 1 ▸, shows N—N and C—S bond distances and S—C—C—S and C—S—C—C torsion angles similar to the values observed in the triclinic form (Li *et al.*, 2011 ▸). As shown by the mol­ecular overlap of the two polymorphs (Fig. 2 ▸), drawn with *Mercury* (Macrae *et al.*, 2008 ▸), there is only a slight difference in their geometry. The tetra­zole rings (N1–N4/C1 and N5–N8/C4) are inclined to one another by 5.50 (6)° in the title polymorph and by 1.9 (2)° in the triclinic polymorph. While there are only small differences in the geometric parameters between the two polymorphic forms, they are enough to produce a different crystal packing.

## Supra­molecular features   

**Figure 3:**
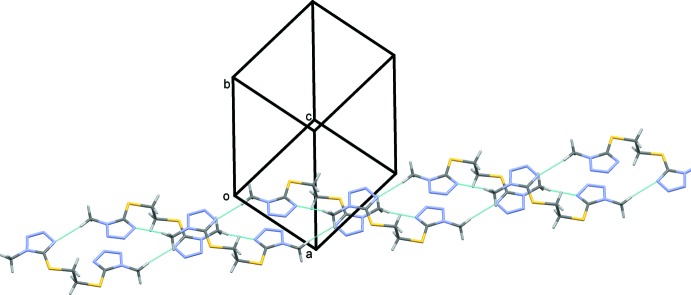
A partial view of the crystal packing of the title compound, showing details of the C—H⋯N hydrogen bonds (dashed lines, see Table 1 ▸).

**Figure 4:**
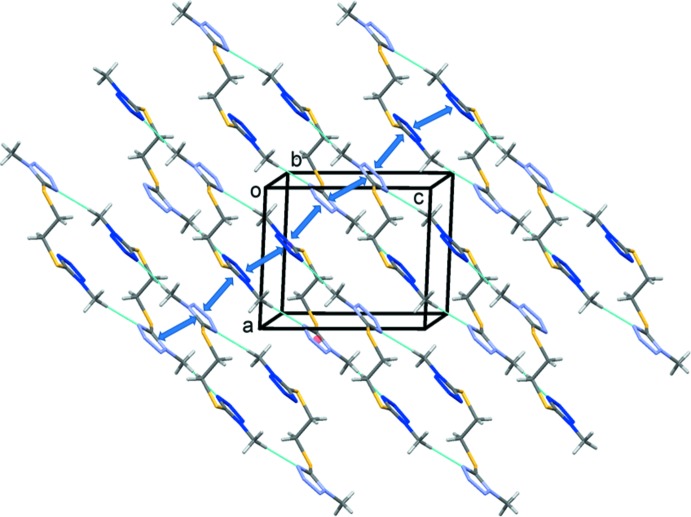
Crystal packing of the title compound, showing details of the C—H⋯N hydrogen bonds (dashed lines, see Table 1 ▸) and examples of the π–π inter­actions (blue double-headed arrows).

In the crystal, mol­ecules are linked by C—H⋯N hydrogen bonds, forming chains propagating along [101] and enclosing 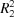
(20) ring motifs (Fig. 3 ▸ and Table 1 ▸). The chains are linked by offset π–π inter­actions involving the tetra­zole rings, forming layers parallel to the *ac* plane, as shown in Fig. 4 ▸. The numerical details of these inter­actions are: *Cg*1⋯*Cg*1^i^ = 3.365 (1) Å, α = 0°, inter­planar distance = 3.2056 (4) Å, offset = 1.024 Å; *Cg*1⋯*Cg*2^ii^ = 3.423 (1) Å, α = 5.5 (1)°, inter­planar distances = 3.278 (4) and 3.321 (4) Å, offset = 0.83 Å; and *Cg*2⋯*Cg*2^iii^ = 3.4227 (7) Å, α = 0°, inter­planar distance = 3.1346 (4) Å, offset = 1.201 Å; *Cg*1 and *Cg*2 are the centroids of the tetra­zole rings N1–N4/C1 and N5–N8/C4, respectively; symmetry codes: (i) −*x* + 1, −*y*, −*z*; (ii) *x* − 1, *y*, *z*; (iii) −*x* + 2, −*y*, −*z* + 1.

As a result of these inter­actions, the mol­ecules are packed very efficiently so that the Kitaigorodskii (1973 ▸) index is 72%. The crystal packing in the crystal of the triclinic polymorph is very similar, with a Kitaigorodskii index of 69% (*PLATON*; Spek, 2009 ▸).

## Database survey   

A search of the Cambridge Structural Database (CSD; version 5.38, last update May 2017; Groom *et al.*, 2016 ▸) for the skeleton of the title compound gave 11 hits. Apart from the crystal structure of the triclinic polymorph of the title compound (CSD refcode EVAWUU; Li *et al.*, 2011 ▸), and that of a diphenyl substituted compound, 1,2-bis­(1-phenyl-1*H*-tetra­zol-5-ylsulfan­yl)ethane (IXAVUY; Wang *et al.*, 2004 ▸), all the others involve coordination compounds of BMTTE.

## Synthesis and crystallization   

The title compound, (BMTTE), was synthesized by a slightly modified version of the procedure described by Li *et al.* (2011 ▸). 5-Mercapto-1-methyl­tetra­zole (9.29 g, 0.08 mol) was added to a solution of sodium hydroxide (3.26 g, 0.08 mol) in EtOH (110 ml). The mixture was stirred at room temperature for one day. Di­chloro­ethane (3.2 ml, 0.04 mol) in 6 ml of EtOH was then added dropwise and the mixture was refluxed for 18 h. The resulting white solid was filtered, washed with H_2_O and dried *in vacuo* (yield 88%; m.p. 417–419 K). Analysis calculated for C_6_H_10_S_2_N_8_: N 43.38, C 27.90, H 3.90%; Found: N 42.31, C 27.85, H 3.28%. IR (cm^−1^): 1469*m*, 1442*m* (1408*m*, 1391*m*) ν(ring); 1276*m*, 1222*m*, ω(CH–CH_2_); 1169*m*, δ(CH); 1144*m*, 1078*m*, 1026*m*, δ(ring); 728*m*, 716*m*, γ(CH); 698*s*, ν(C—S). ^1^H NMR (400 MHz, dmso-*d*_6_) δ in ppm: 3.93 (*s*, 6H, H*b*), 3.66 (*s*, 4H, H*a*). MS–ESI: *m*/*z* (%) = 259 (100) [C_6_H_10_S_2_N_8_+H^+^]. Colourless prismatic crystals were obtained by slow evaporation of a solution in aceto­nitrile.

## Refinement   

Crystal data, data collection and structure refinement details are summarized in Table 2 ▸. The C-bound H atoms were included in calculated positions and treated as riding: C—H = 0.98–0.99 Å with *U*_iso_(H) = 1.5*U*_eq_(C-meth­yl) and 1.2*U*_eq_(C) for other H atoms.

## Supplementary Material

Crystal structure: contains datablock(s) I, Global. DOI: 10.1107/S205698901701341X/su5392sup1.cif

Structure factors: contains datablock(s) I. DOI: 10.1107/S205698901701341X/su5392Isup2.hkl

Supporting information file. DOI: 10.1107/S205698901701341X/su5392Isup3.cdx

Supporting information file. DOI: 10.1107/S205698901701341X/su5392Isup4.cml

CCDC reference: 1575392

Additional supporting information:  crystallographic information; 3D view; checkCIF report

## Figures and Tables

**Table 1:** Hydrogen-bond geometry (Å, °)

*D*—H⋯*A*	*D*—H	H⋯*A*	*D*⋯*A*	*D*—H⋯*A*
C11—H11*B*⋯N8^i^	0.98	2.39	3.3533 (13)	168
C12—H12*B*⋯N4^ii^	0.98	2.36	3.3183 (13)	165

Symmetry codes: (i) 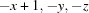; (ii) 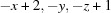.

**Table 2:** Experimental details

Crystal data	
Chemical formula	C_6_H_10_N_8_S_2_
*M*_r_	258.34
Crystal system, space group	Monoclinic, *P*2_1_/*c*
Temperature (K)	100
*a*, *b*, *c* (Å)	8.2456 (10), 13.7471 (17), 9.6878 (12)
β (°)	92.643 (4)
*V* (Å^3^)	1097.0 (2)
*Z*	4
Radiation type	Mo *K*α
μ (mm^−1^)	0.47
Crystal size (mm)	0.25 × 0.22 × 0.19

Data collection	
Diffractometer	Bruker D8 Venture Photon 100 CMOS
Absorption correction	Multi-scan (*SADABS*; Bruker, 2014 ▸)
*T*_min_, *T*_max_	0.697, 0.746
No. of measured, independent and observed [*I* > 2σ(*I*)] reflections	23909, 2725, 2620
*R*_int_	0.024
(sin θ/λ)_max_ (Å^−1^)	0.668

Refinement	
*R*[*F* ^2^ > 2σ(*F* ^2^)], *wR*(*F* ^2^), *S*	0.022, 0.057, 1.08
No. of reflections	2725
No. of parameters	148
H-atom treatment	H-atom parameters constrained
Δρ_max_, Δρ_min_ (e Å^−3^)	0.36, −0.25

Computer programs: *APEX3* (Bruker, 2014 ▸), *SAINT* (Bruker, 2014 ▸), *SHELXS2014* (Sheldrick, 2008 ▸), *Mercury* (Macrae *et al.*, 2008 ▸), *SHELXL2014* (Sheldrick, 2015 ▸), *PLATON* (Spek, 2009 ▸) and *publCIF* (Westrip, 2010 ▸).

## supplementary crystallographic information

### Crystal data

**Table:**

C_6_H_10_N_8_S_2_	*D*_x_ = 1.564 Mg m^−^^3^
*M**_r_* = 258.34	Melting point: 144 K
Monoclinic, *P*2_1_/*c*	Mo *K*α radiation, λ = 0.71073 Å
*a* = 8.2456 (10) Å	Cell parameters from 9507 reflections
*b* = 13.7471 (17) Å	θ = 2.5–28.3°
*c* = 9.6878 (12) Å	µ = 0.47 mm^−^^1^
β = 92.643 (4)°	*T* = 100 K
*V* = 1097.0 (2) Å^3^	Prism, colourless
*Z* = 4	0.25 × 0.22 × 0.19 mm
*F*(000) = 536	

### Data collection

**Table:**

Bruker D8 Venture Photon 100 CMOS diffractometer	2620 reflections with *I* > 2σ(*I*)
φ and ω scans	*R*_int_ = 0.024
Absorption correction: multi-scan (SADABS; Bruker, 2014)	θ_max_ = 28.3°, θ_min_ = 2.5°
*T*_min_ = 0.697, *T*_max_ = 0.746	*h* = −10→11
23909 measured reflections	*k* = −18→18
2725 independent reflections	*l* = −12→12

### Refinement

**Table:**

Refinement on *F*^2^	Secondary atom site location: difference Fourier map
Least-squares matrix: full	Hydrogen site location: inferred from neighbouring sites
*R*[*F*^2^ > 2σ(*F*^2^)] = 0.022	H-atom parameters constrained
*wR*(*F*^2^) = 0.057	*w* = 1/[σ^2^(*F*_o_^2^) + (0.0262*P*)^2^ + 0.493*P*] where *P* = (*F*_o_^2^ + 2*F*_c_^2^)/3
*S* = 1.08	(Δ/σ)_max_ < 0.001
2725 reflections	Δρ_max_ = 0.36 e Å^−^^3^
148 parameters	Δρ_min_ = −0.25 e Å^−^^3^
0 restraints	Extinction correction: (SHELXL2014; Sheldrick, 2015), Fc^*^=kFc[1+0.001xFc^2^λ^3^/sin(2θ)]^-1/4^
Primary atom site location: structure-invariant direct methods	Extinction coefficient: 0.0376 (18)

### Special details

**Table:**

Geometry. All esds (except the esd in the dihedral angle between two l.s. planes) are estimated using the full covariance matrix. The cell esds are taken into account individually in the estimation of esds in distances, angles and torsion angles; correlations between esds in cell parameters are only used when they are defined by crystal symmetry. An approximate (isotropic) treatment of cell esds is used for estimating esds involving l.s. planes.

### Fractional atomic coordinates and isotropic or equivalent isotropic displacement parameters (Å^2^)

**Table:**

	*x*	*y*	*z*	*U*_iso_*/*U*_eq_	
S1	0.46814 (3)	0.19617 (2)	0.21662 (2)	0.01218 (8)	
S2	0.99483 (3)	0.22655 (2)	0.31188 (3)	0.01382 (8)	
N1	0.32833 (10)	0.04027 (6)	0.08604 (8)	0.01158 (16)	
N2	0.33462 (11)	−0.05843 (6)	0.08543 (9)	0.01445 (17)	
N3	0.44707 (11)	−0.08272 (6)	0.17676 (9)	0.01511 (18)	
N4	0.51615 (11)	−0.00275 (6)	0.23918 (9)	0.01383 (17)	
N5	1.15565 (10)	0.07099 (6)	0.42528 (8)	0.01085 (16)	
N6	1.16663 (10)	−0.02693 (6)	0.41075 (9)	0.01411 (17)	
N7	1.06184 (11)	−0.05083 (6)	0.31273 (9)	0.01451 (17)	
N8	0.98081 (11)	0.02894 (6)	0.26071 (9)	0.01352 (17)	
C1	0.44014 (11)	0.07287 (7)	0.18059 (10)	0.01056 (18)	
C2	0.66415 (11)	0.19192 (7)	0.31250 (10)	0.01208 (19)	
H2A	0.6649	0.2392	0.3896	0.014*	
H2B	0.6818	0.1262	0.3520	0.014*	
C3	0.79982 (12)	0.21624 (7)	0.21671 (10)	0.01285 (19)	
H3A	0.7747	0.2784	0.1687	0.015*	
H3B	0.8064	0.1647	0.1458	0.015*	
C4	1.04182 (11)	0.10381 (7)	0.33269 (10)	0.01087 (18)	
C11	0.21707 (12)	0.09470 (7)	−0.00653 (10)	0.0147 (2)	
H11A	0.1360	0.1277	0.0476	0.022*	
H11B	0.1624	0.0499	−0.0721	0.022*	
H11C	0.2781	0.1431	−0.0572	0.022*	
C12	1.25381 (12)	0.12395 (7)	0.52931 (10)	0.0152 (2)	
H12A	1.3335	0.1642	0.4838	0.023*	
H12B	1.3105	0.0776	0.5914	0.023*	
H12C	1.1834	0.1656	0.5827	0.023*	

### Atomic displacement parameters (Å^2^)

**Table:**

	*U*^11^	*U*^22^	*U*^33^	*U*^12^	*U*^13^	*U*^23^
S1	0.01044 (12)	0.00922 (12)	0.01639 (13)	0.00092 (8)	−0.00455 (8)	0.00048 (8)
S2	0.01144 (13)	0.00868 (12)	0.02066 (14)	−0.00105 (8)	−0.00666 (9)	0.00047 (8)
N1	0.0121 (4)	0.0099 (4)	0.0125 (4)	0.0000 (3)	−0.0017 (3)	0.0000 (3)
N2	0.0164 (4)	0.0100 (4)	0.0170 (4)	0.0008 (3)	0.0007 (3)	−0.0005 (3)
N3	0.0153 (4)	0.0120 (4)	0.0178 (4)	0.0002 (3)	−0.0007 (3)	0.0006 (3)
N4	0.0140 (4)	0.0111 (4)	0.0162 (4)	0.0013 (3)	−0.0020 (3)	0.0019 (3)
N5	0.0110 (4)	0.0094 (4)	0.0119 (4)	0.0003 (3)	−0.0017 (3)	0.0007 (3)
N6	0.0156 (4)	0.0100 (4)	0.0168 (4)	0.0010 (3)	0.0019 (3)	0.0010 (3)
N7	0.0161 (4)	0.0115 (4)	0.0159 (4)	−0.0002 (3)	0.0007 (3)	−0.0003 (3)
N8	0.0150 (4)	0.0109 (4)	0.0145 (4)	−0.0013 (3)	−0.0014 (3)	−0.0010 (3)
C1	0.0091 (4)	0.0114 (4)	0.0111 (4)	0.0003 (3)	−0.0007 (3)	0.0007 (3)
C2	0.0107 (4)	0.0114 (4)	0.0136 (4)	0.0000 (3)	−0.0051 (3)	0.0002 (3)
C3	0.0108 (4)	0.0120 (4)	0.0153 (4)	−0.0006 (3)	−0.0048 (3)	0.0015 (3)
C4	0.0095 (4)	0.0115 (4)	0.0114 (4)	−0.0008 (3)	−0.0006 (3)	0.0004 (3)
C11	0.0137 (5)	0.0152 (5)	0.0144 (5)	0.0018 (4)	−0.0056 (4)	0.0008 (4)
C12	0.0152 (5)	0.0157 (5)	0.0140 (4)	−0.0022 (4)	−0.0062 (4)	−0.0003 (4)

### Geometric parameters (Å, º)

**Table:**

S1—C1	1.7438 (10)	N7—N8	1.3681 (12)
S1—C2	1.8276 (10)	N8—C4	1.3290 (12)
S2—C4	1.7409 (10)	C2—C3	1.5232 (14)
S2—C3	1.8218 (10)	C2—H2A	0.9900
N1—C1	1.3461 (12)	C2—H2B	0.9900
N1—N2	1.3578 (12)	C3—H3A	0.9900
N1—C11	1.4594 (12)	C3—H3B	0.9900
N2—N3	1.2956 (12)	C11—H11A	0.9800
N3—N4	1.3663 (12)	C11—H11B	0.9800
N4—C1	1.3278 (12)	C11—H11C	0.9800
N5—C4	1.3459 (12)	C12—H12A	0.9800
N5—N6	1.3569 (12)	C12—H12B	0.9800
N5—C12	1.4580 (12)	C12—H12C	0.9800
N6—N7	1.2964 (12)		
			
C1—S1—C2	100.16 (4)	H2A—C2—H2B	108.2
C4—S2—C3	99.77 (5)	C2—C3—S2	111.39 (7)
C1—N1—N2	108.09 (8)	C2—C3—H3A	109.4
C1—N1—C11	129.71 (8)	S2—C3—H3A	109.4
N2—N1—C11	122.19 (8)	C2—C3—H3B	109.4
N3—N2—N1	106.31 (8)	S2—C3—H3B	109.4
N2—N3—N4	111.44 (8)	H3A—C3—H3B	108.0
C1—N4—N3	105.18 (8)	N8—C4—N5	108.99 (8)
C4—N5—N6	108.14 (8)	N8—C4—S2	127.81 (8)
C4—N5—C12	129.88 (8)	N5—C4—S2	123.14 (7)
N6—N5—C12	121.97 (8)	N1—C11—H11A	109.5
N7—N6—N5	106.38 (8)	N1—C11—H11B	109.5
N6—N7—N8	111.37 (8)	H11A—C11—H11B	109.5
C4—N8—N7	105.12 (8)	N1—C11—H11C	109.5
N4—C1—N1	108.98 (9)	H11A—C11—H11C	109.5
N4—C1—S1	128.32 (8)	H11B—C11—H11C	109.5
N1—C1—S1	122.69 (7)	N5—C12—H12A	109.5
C3—C2—S1	109.91 (7)	N5—C12—H12B	109.5
C3—C2—H2A	109.7	H12A—C12—H12B	109.5
S1—C2—H2A	109.7	N5—C12—H12C	109.5
C3—C2—H2B	109.7	H12A—C12—H12C	109.5
S1—C2—H2B	109.7	H12B—C12—H12C	109.5

### Hydrogen-bond geometry (Å, º)

**Table:**

*D*—H···*A*	*D*—H	H···*A*	*D*···*A*	*D*—H···*A*
C11—H11*B*···N8^i^	0.98	2.39	3.3533 (13)	168
C12—H12*B*···N4^ii^	0.98	2.36	3.3183 (13)	165

Symmetry codes: (i) −*x*+1, −*y*, −*z*; (ii) −*x*+2, −*y*, −*z*+1.
